# Trends in and Correlates of Short-Acting Contraceptive Stock-Outs: Multicountry Analysis of Performance Monitoring for Action Agile Platform Data

**DOI:** 10.9745/GHSP-D-23-00411

**Published:** 2024-06-27

**Authors:** Alain K. Koffi, Pierre Muhoza, Saifuddin Ahmed, Philip Anglewicz, Funmilola OlaOlorun, Elizabeth Omoluabi, Mary Thiongo, Peter Gichangi, Georges Guiella, Pierre Akilimali, P.R. Sodani, Amy Tsui, Scott Radloff

**Affiliations:** a Department of International Health, Johns Hopkins Bloomberg School of Public Health, Baltimore, MD, USA.; b Department of Population, Family and Reproductive Health, Johns Hopkins Bloomberg School of Public Health, Baltimore, MD, USA.; c Department of Community Medicine, University of Ibadan, Ibadan, Nigeria.; d Centre for Research, Evaluation Resources and Development, Ife, Nigeria.; e Statistics and Population Studies Department, University of the Western Cape, Bellville, South Africa.; f International Centre for Reproductive Health Kenya, Nairobi, Kenya.; g Technical University of Mombasa, Mombasa, Kenya.; h Department of Public Health and Primary Care, Faculty of Medicine and Health Sciences, Ghent University, Belgium.; i Institut Supérieur des Sciences de la Population, Joseph Ki-Zerbo University, Ouagadougou, Burkina Faso.; j Ecole de Santé Publique de l’Université de Kinshasa, Kinshasa, Democratic Republic of Congo.; k IIHMR University, Jaipur, India.

## Abstract

Understanding trends in contraceptive stock-outs, as well as their structural and demand-side correlates, is critical for policymakers and program managers to identify strategies to further anticipate, reduce, and prevent stock-outs. We analyzed trends as well as supply- and demand-side correlates of short-acting contraceptive method stock-outs by using data from multiple rounds of Performance Monitoring for Action Agile surveys. These data longitudinally measured contraceptive availability over 2 years (between November 2017 and January 2020) across 2,134 public and private service delivery points (SDPs) from urban areas of 5 countries (Burkina Faso, Democratic Republic of the Congo [DRC], India, Kenya, and Nigeria). For each country, we analyzed the trends and used multilevel mixed-effect logistic regression to model the odds of short-acting contraceptive stock-outs, adjusting for key structural and demand-side factors of the SDPs. Stock-outs in short-acting contraceptive methods were common in health facilities and varied markedly, ranging from as low as 2.9% (95% confidence interval [CI]=1.7%, 5.1%) in India to 51.0% (95% CIs=46.8%, 56.0%) in Kenya. During the observation period, stock-out rates decreased by 28% in the SDP samples in India (aOR=0.72, *P*<.001) and 8% in Nigeria (aOR=0.92, *P*<.001) but increased by 15% in DRC (aOR=1.15; *P=*.036) and 5% in Kenya (aOR=1.05, *P=*.003) with each round of data collection. Correlates of stock-out rates included the facility managerial authority (private versus public), whether the facility was rated high quality, whether the facility was at an advanced tier, and whether there was high demand for short-acting contraceptives. In conclusion, stock-outs of short-acting contraceptives are still common in many settings. Measuring and monitoring contraceptive stock-outs is crucial for identifying and addressing issues related to the availability and supply of short-acting contraceptives.

## Abstract (precis)

Stock-outs of short-acting contraceptives are still common in many settings. Measuring and monitoring contraceptive stock-outs is crucial for identifying and addressing issues related to the availability and supply of short-acting contraceptives.

## Teaser

**Key Findings:**

- As countries pledge to increase access to contraceptives, there is a need for continuous tracking of stock-outs to ensure continuous contraceptive supply and availability.
- The analysis underscores the multifaceted nature of the contraceptive stock-out issue, influenced by both structural supply and demand-side factors that vary across different contexts.

**Key Implications:**

- Policymakers should prioritize the development and implementation of targeted strategies that address the unique challenges of each setting to mitigate contraceptive stock-outs.
- Program managers are encouraged to monitor contraceptive stock-outs to identify when and where they are not available and take steps to ensure that supplies are replenished.
- Donors and international partners have a critical role to play in supporting scalable interventions that address both the structural deficiencies and the demand complexities contributing to contraceptive stock-outs. Funding should also support the expansion of monitoring platforms, such as Performance Monitoring for Action Agile, which are essential for evidence-based decision-making and accountability.

## INTRODUCTION

In many low- and middle-income countries (LMICs), particularly in sub-Saharan Africa, women have limited access to a range of contraceptive methods.^1^ Contraceptive use is driven by a combination of client demand, availability, accessibility, and acceptability of a given method.^2^^,^^3^ Contraceptive stock-outs are defined as the unavailability of “one or more contraceptive options that, routinely or based on policy, should be available at a service delivery point (SDP) ^4^ represent a family planning (FP) access barrier due to the supply chain not functioning effectively.^”^^5^^–^^7^ Contraceptive stock-outs and uneven distribution of contraceptive methods and supplies are frequently cited as key reasons for women’s unmet need for contraception in LMICs and can lead to unintended pregnancies, which also lead to maternal and child health risks, as well as other social and economic consequences.^8^^,^^9^ A recent qualitative study of stock-outs in Uganda that interviewed both clients and providers found that (1) women are accustomed to stock-outs and frequently turn to other providers, often switching to another less effective method; (2) stock-outs lead to negative outcomes for women, such as unintended pregnancy, stress, domestic conflict, and increased overall contraceptive costs; and (3) stock-outs also cause providers to face emotional distress, demoralization, blame, declining skills, and decreased demand for their services.^10^

The World Health Organization has identified addressing contraceptive stock-outs as a key priority for global FP research.^11^ Stock-outs have an impact on contraceptive prevalence and method choice. Significant research suggests that limited options for contraceptive methods have hindered couples’ ability to find a method that meets their specific needs, leading to reduced contraceptive usage rates.^12^^,^^13^ Conversely, the introduction of each new contraceptive method contributed to an increase in overall usage, a trend that was also observed in South Korea, Thailand, and Hong Kong.^14^ Despite the global emphasis on addressing stock-outs, many countries still lack data, and few countries routinely monitor facility-level stock-outs.^15^^,^^16^ The Service Provision Assessment (SPA)^17^ and the Service Availability and Readiness Assessment (SARA) surveys^18^ are standardized health facility assessment tools that are widely used to collect information on service availability and quality-of-care measures for a variety of health services, including FP. SARA surveys are typically conducted approximately every 5 years, whereas SPA surveys are implemented less frequently. The infrequent implementation of these surveys complicates the comparisons of indicators over short periods of time, which in turn limits the ability to understand short-term trends of contraceptive stock-outs and their determinants.

There is a paucity of evidence on the determinants of contraceptive stock-outs, and few studies have systematically assessed the prevalence and trends in contraceptive stock-outs. Many researchers attribute stock-outs to structural and demand-side factors.^12^^,^^19^^–^^22^ Previously studied structural factors include the type of facility offering the contraceptive method, the geographic location or accessibility of the facility,^19^^,^^20^ and the contraceptive method type. Whereas some studies have found contraceptive stock-outs to be more prevalent among public compared with private-sector facilities,^21^ other studies have reported the opposite.^22^ In particular, long-acting contraceptive methods (implants and intrauterine devices) have been shown to be subject to more frequent stock-outs compared with short-acting methods.^23^^,^^24^ The demand-side factors that have been shown to influence contraceptive use include the awareness or accurate knowledge about contraceptive methods,^25^ perceived effectiveness and safety of contraceptive methods,^26^ partner influence,^27^ cultural and social norms,^28^ or individual preferences and needs.^29^^,^^30^

Using a longitudinal dataset that includes multiple rounds of public and private facility-level characteristics as well as stock-out data over 2 years, we aimed to shed light on the trends and structural and demand-side correlates of short-acting contraceptive methods stock-outs in urban or suburban areas of 5 LMICs. This information can help policymakers, program managers, and decision-makers identify strategies to further anticipate, reduce, and prevent stock-outs.

We aimed to shed light on the trends and structural and demand-side correlates of short-acting contraceptive methods stock-outs in urban or suburban areas of 5 LMICs.

## METHODS

### Study Design and Data Sources

Data were drawn from the Performance Monitoring for Action (PMA) Agile study (www.pmadata.org/technical-areas/pma-agile), which was conducted in urban or suburban settings across 5 countries of sub-Saharan Africa and 1 in South Asia. The study design, sampling, and data collection of the PMA Agile project have been described elsewhere.^29^ PMA Agile aimed to: (1) implement a continuous health facility- and client-level data collection system to measure performance indicators in multiple urban or suburban locations to monitor 2 large-scale investments of the Bill & Melinda Gates Foundation to improve marginalized urban or suburban populations’ access to contraceptive services: DKT International and The Challenge Initiative;^31^ and (2) incorporate these data into an indicators dashboard that displays trends in key indicators on the supply, quality, and consumption of FP services. For our purposes, Niger was omitted due to the limited availability of survey data, which comprised only 3 survey rounds only by the time of this analysis. The current study includes a total of 2,134 public and private service delivery points (SDPs) across 13 urban settings in Burkina Faso, Democratic Republic of the Congo (DRC), India, Kenya, and Nigeria that were surveyed 5–6 times over a period of 2 years, roughly corresponding to 2018–2019 with data collection in 3 settings starting in late 2017 and collection in 1 setting continuing into early 2020.^31^

### Measurement: Outcome Variable

PMA Agile collected SDP data on various contraceptive methods, including contraceptive beads, foam/jelly and sterilization, oral pills, intrauterine devices, injectables, male and female condoms, implants, and emergency contraception. The present analysis focused on short-acting methods that are most commonly used in the countries surveyed.^32^ Such methods include injectables, pills, emergency contraception, and female or male condoms. These methods typically require regular administration or application, and therefore, their sustained use requires multiple client–facility interactions each year, which, in turn, highlights the importance of ensuring their availability through regular and timely resupply at facilities. The study outcome was, therefore, a binary variable indicating whether any of the aforementioned short-acting methods of modern contraception were out of stock at the SDP on the day of the survey.

The availability of FP services/products at a given SDP was first determined by the response to the survey question, “Do you usually offer FP services/products?” If the SDP provided FP services/products, the respondent was then asked about the provision of specific contraceptive methods using the survey question, “Which of the following methods are provided to clients at this facility?” The question was followed by a listing of specific contraceptive methods. If the SDP reported offering a given contraceptive method, the enumerator then asked if the method was in stock that day. If yes, the enumerator asked to see the method stock to validate its availability with direct visual observation.

The universal stock-out indicator (i.e., the percentage of facilities stocked out on the day of the assessment with visual validation) served as the outcome variable for this study.^7^

### Measurements: Explanatory Variables

There were 5 rounds of data collection in Burkina Faso and the DRC, and 6 rounds in India, Kenya, and Nigeria. Table 1 shows the specific dates for each round across the study sites. The data collection round in each country was considered an explanatory variable.

**TABLE 1.** Implementation Schedule and Short-Acting Contraceptive Methods Stock-Outs by Urban Study Sites

**Country/Urban or Suburban Site**	**Rounds**	**Dates**	**SDPsSurveyed,No.**	**SDPsOffering AnyShort-ActingMethods, No.**	**Injectables**		**Pills**		**Emergency Contraception**		**Condoms**	
					**SDPsOffering,No.**	**SDPs WithStock-Out,No. (%)**	**SDPs Offering,No.**	**SDPs withStock-Out,No. (%)**	**SDPsOffering,No.**	**SDPs WithStock-Out,No. (%)**	**SDPsOffering,No.**	**SDPs WithStock-Out,No. (%)**
India Ferozabad (Uttar Pradesh)Shikohadbad and Tundla Indore (Madhya Pradesh)Puri (Orissa)	1	Feb 2018–Apr 2018	414	322	77	17 (22)	229	20 (9)	177	11 (6)	253	13 (5)
	2	Jul 2018–Aug 2018	394	331	75	11 (15)	255	23 (9)	225	11 (5)	269	11 (4)
	3	Nov 2017–Jan 2018	369	324	59	7 (12)	228	11 (5)	199	8 (4)	256	11 (4)
	4	Feb 2019–May 2019	359	321	46	4 (9)	191	11 (6)	171	12 (7)	209	6 (3)
	5	Jun 2019–Aug 2019	352	322	45	2 (4)	195	7 (4)	194	7 (4)	212	8 (4)
	6	Sep 2019–Dec 2019	279	260	32	2 (6)	135	6 (4)	134	3 (2)	146	4 (3)
Burkina Faso Ouagadougou Koudougou	1	Mar 2018–May 2018	269	155	88	19 (22)	123	6 (5)	41	4 (10)	110	7 (6)
	2	Aug 2018–Oct 2018	262	151	83	11(13)	113	3 (3)	37	1 (3)	105	8 (8)
	3	Feb 2019–Apr 2019	269	157	88	12 (14)	122	11 (9)	38	1 (3)	116	4 (3)
	4	Jun 2019–Sep 2019	222	147	91	15 (16)	114	7 (6)	34	1 (3)	101	1 (1)
	5	Oct 2019–Nov 2019	246	152	80	6 (8)	118	6 (5)	40	1 (3)	107	2 (2)
DRC Kinshasa	1	Dec 2017–Jan 2018	200	154	85	20 (24)	92	23 (25)	77	10 (13)	131	10 (8)
	2	Mar 2018–Jun 2018	197	155	91	25 (27)	109	23 (21)	84	31 (37)	138	6 (4)
	3	Sep 2018–Nov 2018	189	150	90	37 (41)	114	27 (24)	85	31 (36)	125	12 (10)
	4	Feb 2019–Apr 2019	186	151	98	38 (39)	108	38 (35)	89	34 (38)	135	12 (9)
	5	Jun 2019–Aug 2019	184	150	103	37 (36)	119	38 (32)	80	34 (43)	140	12 (9)
Kenya Kericho Migori Uasin Gishu	1	Nov 2017–Jan 2018	618	517	450	86 (19)	450	102 (23)	257	104 (40)	494	65 (13)
	2	Mar 2018–Augt 2018	594	530	481	101 (21)	467	102 (22)	283	72 (25)	509	26 (5)
	3	Oct 2018–Dec 2018	577	534	468	89 (19)	473	93 (20)	286	53(19)	526	17 (3)
	4	Feb 2019–Jun 2019	573	546	497	114 (23)	495	82 (17)	302	56 (19)	531	38 (7)
	5	Jul 2019–Sep 2019	591	530	468	98 (21)	467	85 (18)	276	54 (20)	517	35 (7)
	6	Oct 2019–Jan 2020	593	545	464	118 (25)	490	71 (14)	293	54 (18)	531	31 (6)
Nigeria Kano Lagos Ogun	1	Nov 2017–Jan 2018	633	473	367	85 (23)	361	66 (18)	90	17 (19)	360	70 (19)
	2	Mar 2018–Aug 2018	609	572	481	107 (22)	447	113 (25)	109	18 (17)	437	95 (22)
	3	Sep 2018–Nov 2018	603	593	491	127 (26)	497	85 (17)	104	28 (27)	451	99 (22)
	4	Feb 2019–May 2019	588	582	477	123 (26)	422	54 (13)	111	19 (17)	414	71 (17)
	5	Jun 2019–Aug 2019	584	527	423	120 (28)	378	55 (15)	117	11 (9)	373	66 (18)
	6	Sep 2019–Nov 2019	576	513	405	120 (30)	378	28 (7)	112	14 (13)	364	33 (9)

Abbreviations: DRC, Democratic Republic of the Congo; SDP, service delivery point.

The managerial authority variable described private and public SDPs in each country.

Considering that the quality of FP services could be multifaceted and context specific, dimensionality reduction approaches were used to build a composite measure of FP service quality for the SDPs.^33^^,^^34^ The construction of our quality index was informed by structural health facility determinants such as staffing levels, management practices, and the availability of materials and equipment, which have been shown to be critical to the quality of FP services.^35^ The resulting composite measure included (1) total number of facility staff trained to provide FP services, (2) whether an SDP supported community health volunteers, (3) number of days per week during which FP services were offered, (4) whether a facility had received any supportive supervision in the past 3 months, (5) whether water and electricity were available on the day of the survey, (6) whether fees were charged for FP services, (7) total number of contraceptive methods offered at the SDP, (8) whether the SDP offered long-acting reversible contraceptive methods, and (9) whether the facility referred FP clients to other health care facilities (including counseling and consultation as well as provision/sale of FP methods). The staffing variable was transformed to log scale to ensure normality and included all health care personnel, their titles differing by country.

Principal component analysis was used to construct the composite measure of FP service quality by using the 9 variables described previously. Variables were normalized and coded with similar valence (e.g., larger values for continuous variables, and higher categories for categorical variables were associated with higher quality). The same variables were included for all countries unless they were not available. The factor loadings for the first principal component that explained the highest proportion of the variance in each SDP sample were used as weights to calculate a normalized SDP quality score. The alpha coefficients for the items were considered acceptable^36^ and were Burkina Faso (0.70), Kenya (0.68), DRC (0.57), India (0.52), and Nigeria (0.48). The SDP quality score was categorized into tertiles, indicating low, medium, and high quality.

In this study, hospitals, health centers, and clinics were considered as advanced facilities, whereas pharmacies and drug shops were not. Advanced facilities, unlike pharmacies and drug stores, provide a wide range of comprehensive and personalized contraceptive services, including counseling, diagnosis, and treatment of health conditions that may affect contraceptive choice or use.

The lagged proportion of client volume was generated as a proxy to the demand**-**side variable at each subsequent round and consisted of the difference between the proportion of client volume for the short-acting methods (i.e., injectables, pills, emergency contraception, and female or male condoms) relative to the overall client volume (for all methods) at the indexed round, and that of following one. More specifically, the lagged proportion ($P_{n})$ at a given Round n is:

$$P_{n}=R_{n}-R_{n+1}$$

where $R_{n}$ is the proportion of client volume for the short-acting methods relative to the overall client volume at a round n and is estimated by this formula:

$$R_{n}=\frac{\alpha_{n}inject+\beta_{n}pills+\partial_{n}emerg+\vartheta_{n}cond}{\theta_{all}}$$

where $\alpha_{n}inject$ represents injectable specific client volume at round n, $\beta_{n}pills$ represents pills specific client volume at round n, $\partial_{n}emerg$ represents emergency contraceptive specific client volume at round n, $\vartheta_{n}cond\;$represents female and male condoms specific client volume at round n, $\theta_{all}$ represents the total client volume for any modern contraceptive methods offered at round n, n represents the round of data collection, from 1 to 5 or 6, depending on the study site.

### Statistical Analysis

The trends of short-acting stock-out rates (and 95% confidence interval [CI]) were plotted for 5–6 rounds per study site (over approximately 2 years). We first examined graphically whether short-acting method stock-out rates may change linearly or nonlinearly. Given the hierarchical structure in measuring the outcome variable over rounds of data collection and nested within SDPs, linear and quadratic multilevel mixed-effect logistic regression models were specified.^37^ According to the principle of parsimony, model selection methods should value both descriptive accuracy and simplicity. Thus, we included the round variable in the linear model as a linear function, and in the quadratic model, an additional square term of the survey round variable was added. In instances in which the quadratic term was not statistically significant (*P*>.05), we considered the trend as linear, adhering to the principle of parsimony in our model selection.^38^ We also tested the linear trends for linear and quadratic models using a non-parametric trend test and reported on the corresponding Z and *P*-value.^39^

Predictive models were developed to understand whether the same set of explanatory variables could also be used to predict stock-outs. An advantage of these model specifications is that the round rate of change in stock-out rates could be estimated from the average marginal effect of the round.^40^ Thus, the average marginal effect that approximately averaged the slopes of the change in stock-out prevalence rates was estimated across all data points over the study period. From these predictive models, we derived the conditional expected values of the stock-out rates for variables, such as rounds of surveys and facility quality measure, by differentiating dy/dx (that is, the difference in the dependent variable Y for a change in the explanatory variable X [regressor], along with the significance [*P*-value] of the change). These margin results and the stock-out prevalence rates were then displayed graphically, following the models.

All data analysis was conducted with Stata version 16.0.0 (Stata Corporation, College Station, TX, USA).

### Ethical Approval

Agile data collection protocols were reviewed and approved by the Johns Hopkins Bloomberg School of Public Health Institutional Review Board and the in-country counterpart review board: Kenyatta National Hospital-University of Nairobi Ethics Research Committee; National Health Research Ethics Committee of Nigeria; Ministry of Health (MOH)-Burkina Comité d’Ethique pour la Recherche en Santé; University of Kinshasa School of Public Health Institutional Review Board; and Indian Institute for Health Management Research Ethical Review Board. All participants provided consent in accordance with country-specific approved consent procedures before participating in the survey.

## RESULTS

Table 1 shows an overview of the SDPs surveyed, short-acting contraceptive methods stock-out rates in each study site by year and round. Stock-out rates fluctuated by method, across countries, and between rounds. Across countries, stock-outs for injectables were prevalent.

**FIGURE 1:**
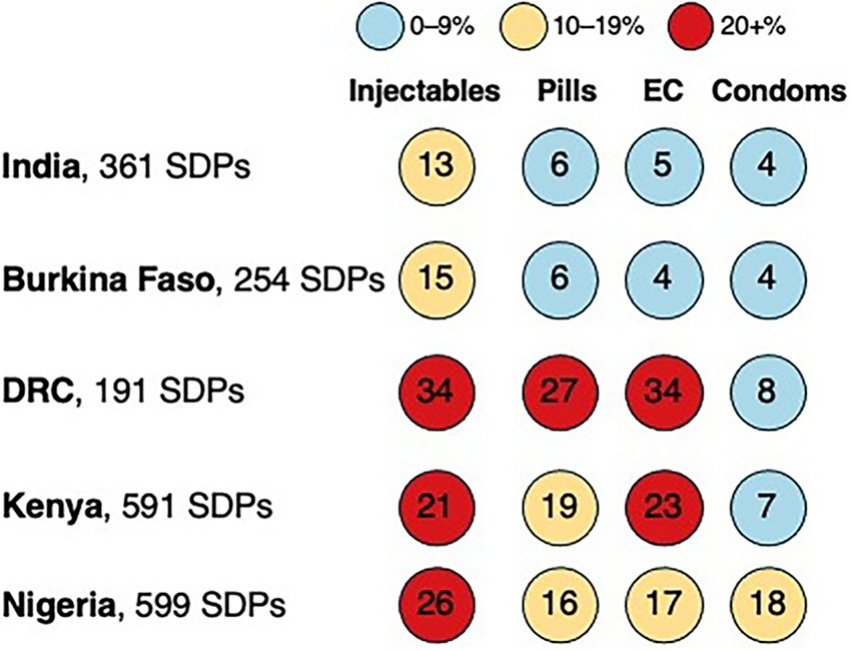
Percentage of Urban/Suburban SDPs Stocked Out of Method Among Facilities That Typically Offer That Method^a^ Abbreviations: DRC, Democratic Republic of the Congo; EC, emergency contraception; SDP, service delivery point. ^a^ Stock-out percentages are averaged across survey rounds and across country settings.

Figure 1 depicts the percentage of SDPs stocked out of each short-acting method, averaged across survey rounds for each country setting, also drawing upon data from Table 1. Both India and Burkina Faso had 3 of the 4 short-acting methods with fewer than 10% stock-out, with only injectables being stocked out at higher levels but still less than 15% stock-outs. Stock-outs were a more serious problem in the facilities tracked in the 3 other country settings. For DRC and Kenya, only condoms were below 10% stock-out levels—the remaining 3 methods failed to meet this mark and by large margins. Facilities experienced stock-outs ranging from 19%–23% and 27%–34% for these 3 methods, respectively, in Kenya and DRC. For Nigeria, all 4 short-acting methods were stocked out at levels ranging from 16%–26% of facilities.

Stock-outs were a more serious problem in DRC, Kenya, and Nigeria for 3 of 4 short-acting methods.

**FIGURE 2:**
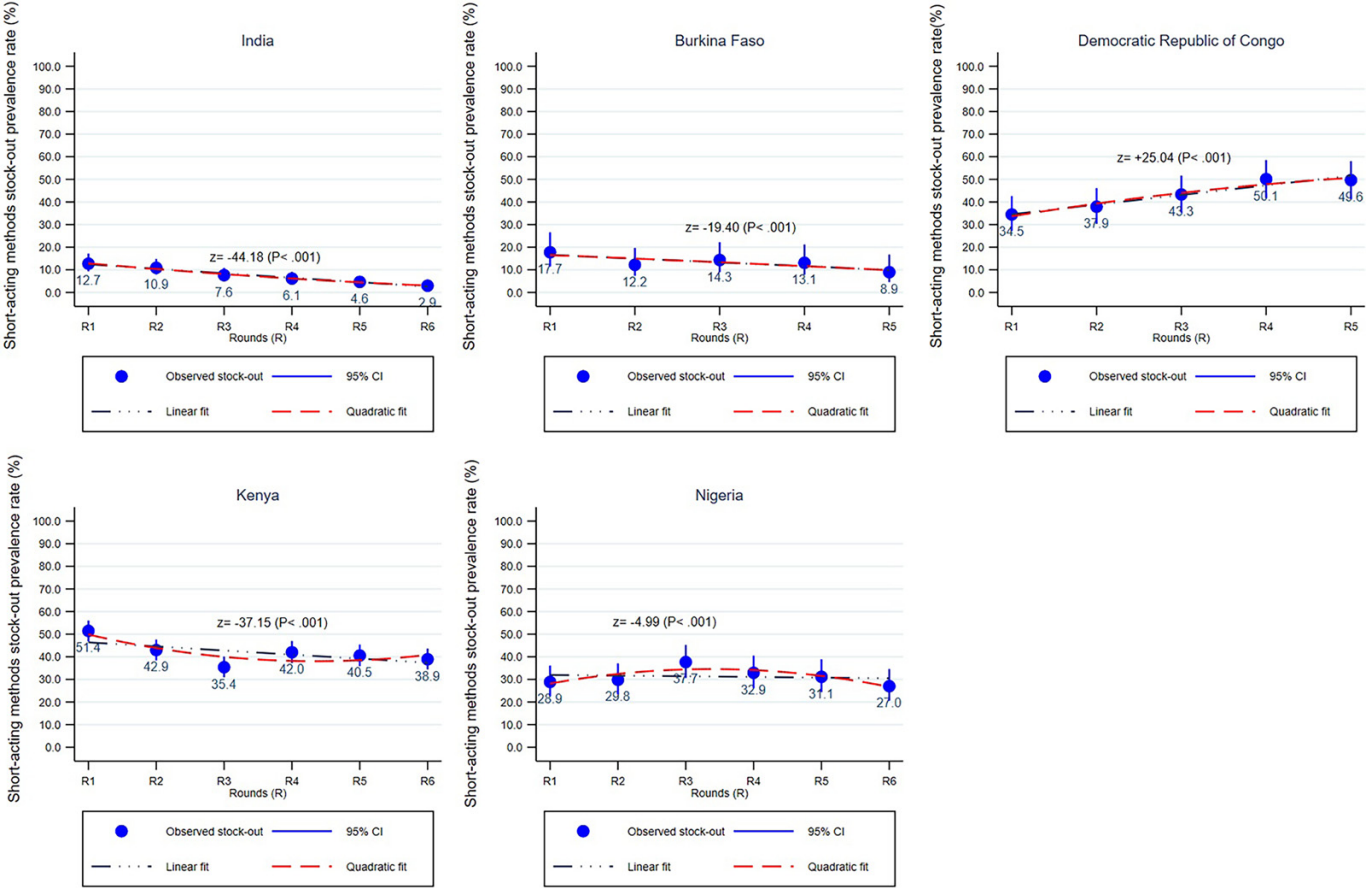
Trends in Short-Acting Methods^a^ Stock-Out Rates (and 95% CI) at Urban/Suburban SDPs, by Round and by Country Abbreviations: CI, confidence interval; SDP, service delivery point. ^a^ Short-acting methods include injectable, pills, emergency contraception, female, or male condoms. nptrend tests for linear trend across ordered groups (study rounds) as reported by Z and *P*-values.

Taking a more detailed look at stock levels between multiple survey rounds reveals substantial variation and stock-out rate fluctuation, from as low as 2.9% (95% CI=1.7%, 5.1%) at Round 6 in India to 51.0% (95% CI=46.8%, 56.0%) at Round 1 in Kenya. In DRC, there was a general increasing trend (z=+25.04, *P*<.001) each round for stock-out rates from 34.5% in Round 1 (95% CI=27.2%, 42.6%) to 49.6% in Round 5 (95% CI=41.3%, 58.0%), whereas there was a linear downward trend (z=−19.40, *P*<.001) in Burkina Faso (17.7%, 95% CI=11.4%, 26.6%; to 8.9%, 95% CI=4.6%, 16.7%) and India (12.7%, 95% CI=9.3%, 17.1%; to 2.9%, 95% CI=1.7%, 5.1%) (Figure 2). Stock-out rates in Kenya and Nigeria exhibited fluctuations with a quadratic trend, suggesting a quadratic model, compared with a linear one, would better fit the multivariate analysis.

Table 2 shows the multilevel mixed-effect regression models of short-acting contraceptive stock-out by explanatory variables and country. In Burkina Faso and India, the adjusted odds of stock-out significantly decreased by round. With each round of data collection, the stock-out odds decreased by 15% in Burkina Faso (aOR=0.85, *P=*.065) and by 28% in India (aOR=0.72, *P*<.001). Similarly, the adjusted odds of stock-out also decreased by 8% in Nigeria (aOR=0.92, *P*<.001). Conversely, they significantly increased at each round of data collection by 15% in DRC (OR=1.15; *P=*.036) and 5% in Kenya (aOR=1.05, *P=*.003). Compared with public facilities, private facilities were significantly associated with increased odds of stock-out in Burkina Faso and Nigeria and decreased odds in DRC (aOR=0.31, *P=*.022), India (aOR=0.10, *P*<.001), and Kenya (aOR=0.58, *P=*.001).

**TABLE 2.** Mixed-Effects Logistic Regression of Short-Acting Method Stock-Out at Urban/Suburban SDPs, by Country

	**India**		**Burkina Faso**		**Democratic Republic of the Congo**		**Kenya**		**Nigeria**	
	aOR	*P* Value	aOR	*P* Value	aOR	*P* Value	aOR	*P* Value	aOR	*P* Value
Round	0.72^a^	.000^a^	0.85	.065	1.15^a^	.036^a^	0.65^a^	.000^a^	1.71^a^	.000^a^
Round ^2		** **					1.05^a^	.003^a^	0.92^a^	.000^a^
Management authority										
Public	1.00	–	1.00	–	1.00	–	1.00	–	1.00	–
Private	0.10^a^	.000^a^	2.90^a^	.016^a^	0.31^a^	0.022^a^	0.58^a^	.001^a^	1.86^a^	.003^a^
Facility quality measure										
Low	1.00	–	1.00	–	1.00	–	1.00	–	1.00	–
Medium	2.63	.007^a^	1.07	0.873	2.46^a^	.002^a^	1.15	.385	1.78^a^	.001^a^
High	5.42	.006^a^	3.23^a^	.016^a^	6.20^a^	.000^a^	0.67^a^	.036^a^	1.54^a^	.043^a^
Advanced facility		** **				** **		** **		
No	1.00	–	1.00	–	1.00	–	1.00	–	1.00	–
Yes	0.30^a^	.049^a^	3.68^a^	.005^a^	0.15^a^	.001^a^	2.14^a^	.000^a^	0.85	.516
Lagged client volume	2.52^a^	.033^a^	1.73	.215	1.22	.434	0.97	.823	1.09	.527
Models diagnostics										
Number of observations	1,847		742		742		3,162		3,207	
Number of groups	334		168		167		539		561	
Wald chi2(6)	58.8		21.86		34.84		97.20		35.8	
*P* value	.000		.001		.000		.000		.000	

Abbreviation: aOR, adjusted odds ratio.

^a^ Statistically significant aORs and *P*-values (<.05).

Adjusting for client demand and other correlates, high-quality SDPs in Burkina Faso, DRC, India, and Nigeria were significantly more likely to experience stock-out in short-acting methods relative to low- and medium-quality SDPs. Conversely, in Kenya, the odds of high-quality SDPs experiencing stock-outs compared with low-quality SDPs were 33% lower (aOR=0.67, *P*=.036).

Advanced SDPs were significantly associated with increased odds of stock-out in Burkina Faso (aOR=3.68, *P*=.005) and Kenya (OR=2.14, *P*<.001) but with a decreased odds in DRC (aOR=0.15, *P*=.001) and India (aOR=0.30, *P*=.049). There was no statistically significant difference by advanced SDP status in Nigeria (aOR=0.85, *P*=.516).

Facilities with high client volume were more likely to face stock-outs in short-acting contraceptive methods in all countries except Kenya. In India, for example, facilities with high client volume were significantly and more prone to stock-out compared with low volume (aOR=2.52, *P*<.001).

**FIGURE 3:**
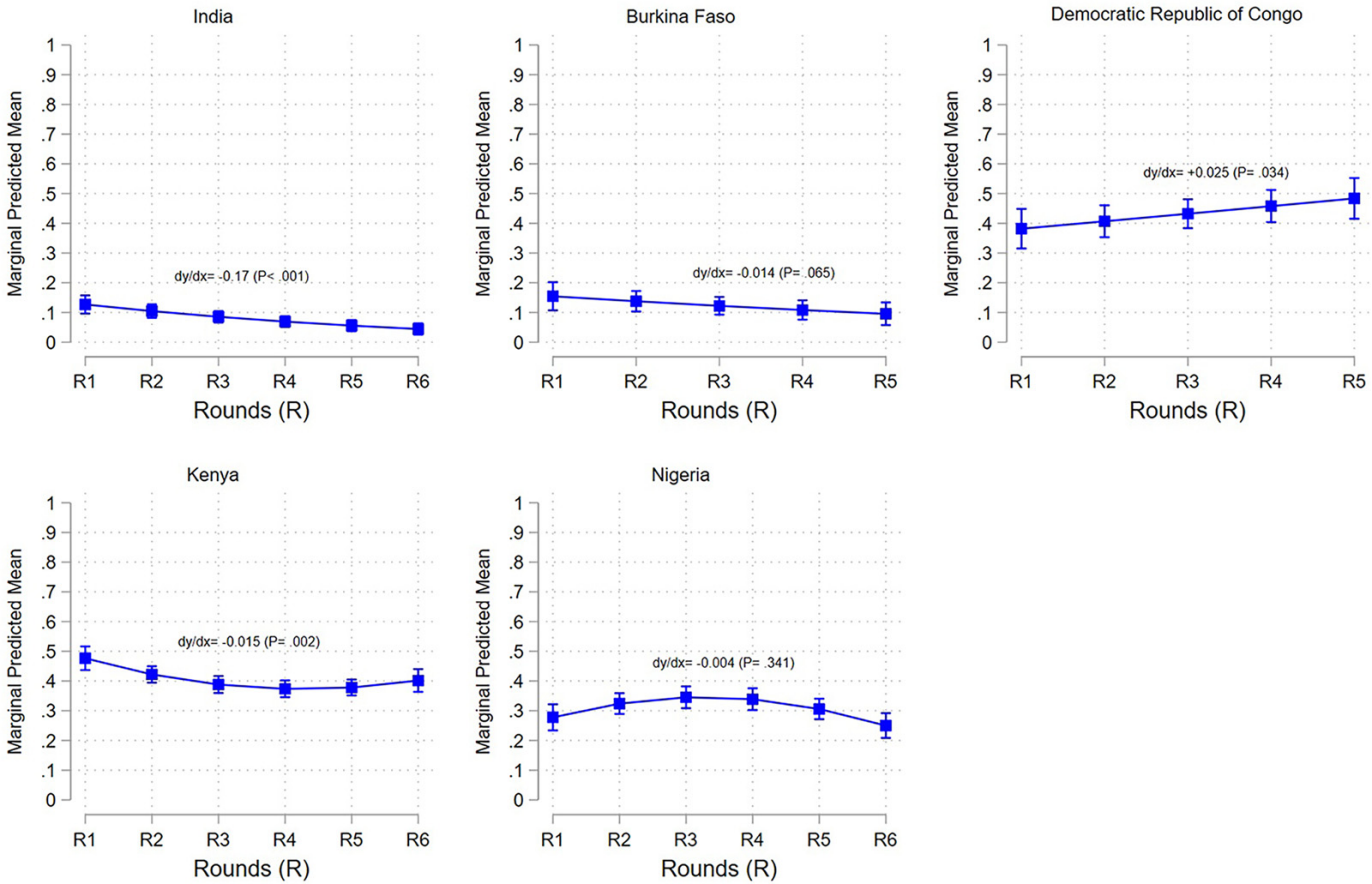
Marginal Predictive Mean of Short-Acting Method Stock-Out With 95% CI at Urban/Suburban SDPs, by Country Abbreviations: CI, confidence interval; SDP, service delivery point.

**FIGURE 4:**
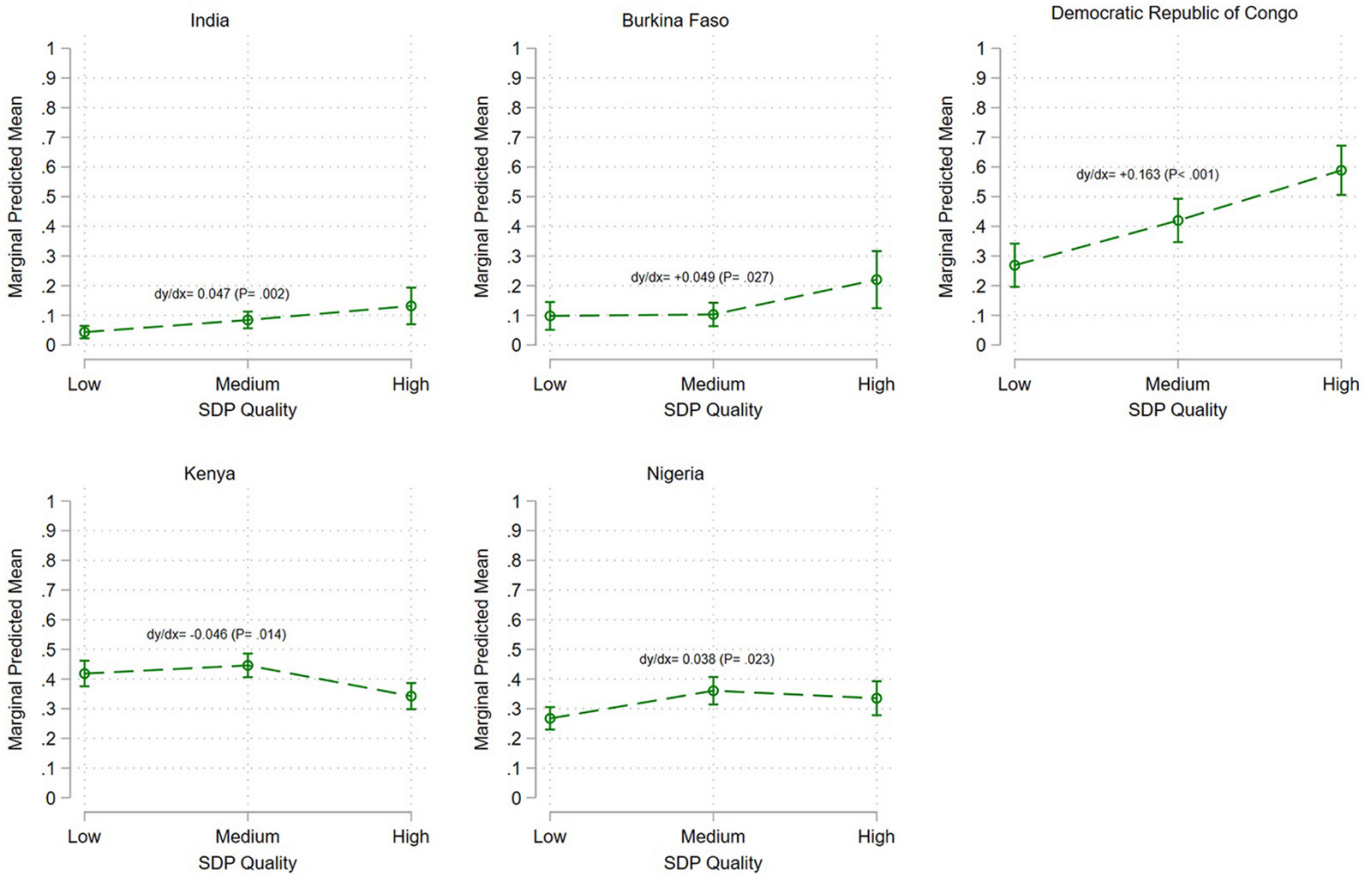
Marginal Predictive Mean of Short-Acting Method Stock-Out With 95% CI at Urban/Suburban SDPs, by SDP Quality and by Country Abbreviations: CI, confidence interval; SDP, service delivery point.

Figure 3 indicates a linear downward trend in marginal predicted means of stock-out rates in Burkina Faso from 15.5% (95% CI=10.7%, 20.2%) in Round 1 to 9.5% (95% CI=5.7%, 13.4%) in Round 5, albeit not significantly (*P*=.065), and in India from 12.7% (95% CI=9.6%, 15.7%) in Round 1 to 4.4% (95% CI=5.7%, 13.4%) in Round 6 [dy/dx=−0.17 (*P*<.001)], after controlling for other covariates. Conversely, Figure 2 indicates a linear upward trend in DRC from 38.2% points at Round 1 (95% CI=31.5%, 44.9%) to 48.4% (95% CI=41.5%, 55.2%) and a quadratic trend in Kenya as well as Nigeria. In all other countries except Kenya, the marginal predicted means of stock-out rates increased from low- to high-quality SDPs (Figure 4).

## DISCUSSION

Stock-outs have an impact on contraceptive prevalence and method choice, and reducing contraceptive stock-outs is a critical measure of success as per Family Planning 2020 (FP2020) and its successor, FP2030. Yet, little is known about patterns of contraceptive stock-outs in LMICs, primarily due to the low frequency of data collection and data limitations. In this study, we focused on the trends in and the operational drivers of short-acting contraceptive methods stock-outs at urban/suburban SDPs in 5 countries: Burkina Faso, DRC, India, Kenya, and Nigeria. The data we used from the PMA Agile study are well suited for this analysis because they include measures of stock-outs and factors that likely impact contraceptive supply. Furthermore, because the data were collected multiple rounds each year across both public and private facilities in each setting, they provide a unique opportunity for a detailed assessment of the trends and determinants of contraceptive stock-outs.

Overall, we found that stock-outs of short-acting contraceptives were common, with fluctuations across rounds of data collection and countries.^21^ Short-acting contraceptive stock-out rates were lower (<20.0%) across rounds in Burkina Faso and India compared with those observed in the other countries included in the study. This finding may suggest a well-functioning supply chain and funding level for contraceptive commodities in those 2 countries. This is not so surprising for India, given its middle-income economy and many years of supply chain investments. It is perhaps more remarkable in Burkina Faso because their prioritization of FP in their public health program is much more recent.^41^ Burkina Faso was host to the 2011 Ouagadougou Conference, where 9 countries in West Africa committed to elevating attention and resources to expanding access to family planning services, soon thereafter forming the Ouagadougou Partnership. Among the 9 Ouagadougou Partnership countries, Burkina Faso has experienced a rapid increase in access/use of contraceptives (with modern contraceptive use increasing nearly 2 percentage points per year from 16%–28% between 2014 and 2022).^41^ Further investigation may be warranted at the country level to understand and explain the decrease in stock-outs.

Across all countries in the study except Kenya, SDPs with high client volume or of high quality (as opposed to SDPs with low client volume or low quality, respectively) were more likely to experience stock-outs in short-acting contraceptives, significantly more so in India. Although this may seem counterintuitive, some considerations could potentially explain this phenomenon. It is possible that high-quality SDPs may attract more clients relative to SDPs with suboptimal quality if they are perceived to provide superior care or operate more efficiently. This could result in an increased demand or a faster consumption of stock that could strain their stock levels, leading to a higher likelihood of experiencing stock-outs, especially if contraceptive supply chains and associated staffing are not adequately managed to meet the heightened demand. In Tumlinson et al.,^42^ the authors found that facilities experienced stock-outs because providers were not completing documentation appropriately and failing to order the commodities they needed. Other reasons for stock-out include the challenges in forecasting their family planning needs and not knowing if or when the order would come.^42^

Across all countries in the study except Kenya, SDPs with high client volume or of high quality were more likely to experience stock-outs in short-acting contraceptives, significantly more so in India.

Compared with public SDPs, the odds of short-acting contraceptive stock-outs were significantly lower for private SDPs in DRC (aOR=0.31, *P=*.022), India (aOR=0.10; *P*<.001), and Kenya (aOR=0.58, *P=*.001). The private health sector is an important source of health services, including reproductive health, in these 3 countries.^43^^,^^44^ Private facilities generally score higher than public facilities in operational capacity.^44^ On average, private facilities are more likely than public facilities to have basic infrastructure and logistics and larger supply chains that often use their own procurement agencies, warehouses, and distribution systems.^45^

### Limitations

This study has several limitations. First, by focusing on urban settings in each of these countries, this study is unable to address the situation of rural SDPs where contraceptive stock-out may be acute. Expanding this research to rural SDPs is warranted to identify challenges contributing to contraception stock-outs that may lead to unmet need. Second, the alpha coefficients for the items used to generate the SDP quality score ranged from 0.48 in Nigeria to 0.70 in Burkina Faso. These may look relatively poor to acceptable internal consistency and, hence, may be indicative of a problem with the score generated.^46^^,^^47^ However, Schmitt suggested that there is no general level where alpha becomes acceptable, but rather, an alpha coefficient of low value can still prove useful in some circumstances.^48^ Furthermore, there is little information on the impact of contraceptive stock-outs on women, their families, and service providers. In the literature, contraceptive stock-outs have been cited as a factor that limits women’s ability to access contraceptives,^12^ but there was no direct information assessing how or to what extent such stock-outs impact women’s access to and use of contraception. In many cases, it was also not clear what methods should be available (based on policy or provider skill/training) at a specific facility or facility type. Further research on the impact of stock-outs on women, families, health care providers, and communities is needed to better inform strategies to mitigate harmful effects of stock-outs, as well as to mobilize political will and financial resources to reduce their occurrence. In addition, the composite measure generated for facility FP service quality may have led to some information loss. Future research should examine the relative influence of the different individual characteristics on contraceptive stock-outs. As this is a secondary analysis, we were restricted to the variables collected at the SDP level within the scope of the initial study, and thus, we may not have fully captured all relevant nuances related to the complex issue of contraceptive stock-outs. For instance, the PMA Agile project did not collect details related to supply chain operations or contextual information on policy or financial determinants of contraceptive availability. The incorporation of such information would have enriched our analysis. Future studies should incorporate these considerations in their analyses for an improved understanding of the complexities of contraceptive stock-outs.

## Notes

**Peer Reviewed**

**First published online:** May 14, 2024
